# Protein Loading into Spongelike PLGA Microspheres

**DOI:** 10.3390/pharmaceutics13020137

**Published:** 2021-01-21

**Authors:** Yuyoung Kim, Hongkee Sah

**Affiliations:** 1 College of Pharmacy, Ewha Womans University, 52 Ewhayeodaegil, Seodaemun-gu, Seoul 03760, Korea; kyy5414@donga.co.kr; 2 Pharmaceutical Product Research Laboratories, Dong-A ST R&D Center, 21, Geumhwa-ro 105beon-gil, Giheung-gu, Yongin-si, Gyeonggi-do 17073, Korea

**Keywords:** poly-d,l-lactide-*co*-glycolide, microencapsulation, porous microspheres, open-pore, closed-pore, protein

## Abstract

A self-healing microencapsulation process involves mixing preformed porous microspheres in an aqueous solution containing the desired protein and converting them into closed-pore microspheres. Spongelike poly-d,l-lactide-*co*-glycolide (PLGA) microspheres are expected to be advantageous to protein loading through self-healing. This study aimed to identify and assess relevant critical parameters, using lysozyme as a model protein. Several parameters governed lysozyme loading. The pore characteristics (open-pore, closed-pore, and porosity) of the preformed microspheres substantially affected lysozyme loading efficiency. The type of surfactant present in the aqueous medium also influenced lysozyme loading efficiency. For instance, cetyltrimethylammonium bromide showing a superior wetting functionality increased the extent of lysozyme loading more than twice as compared to Tween 80. Dried preformed microspheres were commonly used before, but our study found that wet microspheres obtained at the end of the microsphere manufacturing process displayed significant advantages in lysozyme loading. Not only could an incubation time for hydrating the microspheres be shortened dramatically, but also a much more considerable amount of lysozyme was encapsulated. Interestingly, the degree of microsphere hydration determined the microstructure and morphology of closed-pore microspheres after self-healing. Understanding these critical process parameters would help tailor protein loading into spongelike PLGA microspheres in a bespoke manner.

## 1. Introduction

To date, the US FDA has approved 12 long-acting poly-d,l-lactide-co-glycolide (PLGA) microsphere products for the treatments of endocrine disorders, cancer, periodontitis, and osteoarthritis. It is proven that extended-release formulations of PLGA microspheres enhance drug safety and efficacy and improve patient compliance by minimizing dosing frequency. The majority of these microspheres are commercialized through emulsion-based manufacturing processes, and they have nonporous and compact matrices. It may suggest that the inside of PLGA microspheres made by a water-in-oil-in-water (w_1_/o/w_2_) double emulsion method contain macropores [1,2]. Strictly speaking, they are just empty voids that remain as w_1_ droplets are removed from the microspheres by drying.

Despite the great success of PLGA microspheres as depot formulations of peptides and hydrophobic low-molecular-weight drugs, there are no commercial long-acting PLGA microspheres for biomacromolecules such as polypeptides, proteins, bacterial/viral antigens, and polynucleotides. The complexity of their thermodynamic and kinetic stability presents a significant challenge to developing PLGA microsphere formulations. For example, during microsphere manufacturing processes, biomacromolecules are exposed to harsh environments such as heat, shear force, cavitation, organic solvent, or water-organic solvent interface. In particular, proteins tend to be denatured and aggregated during typical emulsion-template microencapsulation processes. Therefore, an adequate strategy is needed to overcome these hurdles [3]. For instance, Diana et al. first formulated human growth hormone (hGH) into hGH-dextran particles, to preserve its structural integrity during a w_1_/o/w_2_ double emulsion process [4]. Interactions between biomacromolecules and the hydrophobic matrix or carboxyl end groups of PLGA microspheres also have a wide array of ramifications in the stability of biomacromolecules. Lastly, autocatalysis of PLGA generates an acidic microenvironment to drive chemical degradation of biomacromolecules inside microspheres [5,6].

Recently, porous microspheres have found wide applications in delivering drugs and biomacromolecules [7,8,9,10,11,12,13,14]. They also are excellent adsorbents and further processed into scaffolds for tissue engineering and regenerative medicine [15,16,17,18,19]. Our study relates to the application of porous PLGA microspheres as a long-acting depot for biomacromolecules. PLGA is an intrinsic self-healing or self-repairing polymer that shows its specific performance (e.g., inter-diffusion, entanglements, and physical interactions of polymer chains) against external stimuli such as temperature, pH, organic solvent, infrared irradiation, and an additive. Schwendeman’s research group pioneered developing the microencapsulation of biomacromolecules into preformed porous PLGA microspheres through a self-healing process [20,21]. Dried PLGA microspheres with an open pore structure were suspended in an aqueous biomacromolecule solution. When the aqueous phase temperature was increased above the glass-transition temperature (Tg) of PLGA, the microsphere pores became closed due to the rearrangements of polymeric chains. This self-healing microencapsulation process allowed loading biomacromolecules into PLGA microspheres without damage to their structural integrity. Similarly, methoxypoly(ethylene glycol)-*b*-poly-lactide (PELA) also was utilized as a porous microsphere-forming material [22]. Porous PELA microspheres prepared using a w_1_/o/w_2_ double emulsion technique were treated with organic solvent or infrared irradiation to close pores in the microsphere surface. During this self-healing process, proteins or latex particles could be loaded into the resultant closed-pore microcapsules. These findings became a catalyst for a series of follow-up studies [23,24,25,26].

Our previous study enabled us to prepare spongelike PLGA microspheres with extreme porosity by a new ammonolysis-based single emulsion microencapsulation technique [27]. It is anticipated that the spongelike microspheres might be advantageous to the loading of biomacromolecules. At present, there remains a need for a better understanding of the self-healing microencapsulation process and its critical process parameters. Investigated in this study were identifying and assessing such essential parameters of the process influencing protein encapsulation. Relevant examples included microsphere porosity, microsphere pore structure (open-pore v. closed-pore), the surfactant type and its aqueous concentration, aqueous protein concentration, and the degree of microsphere hydration. So far, dried preformed PLGA microspheres have been frequently used for protein loading through the self-healing process. However, our study evaluated the applicability of wet microspheres obtained at the end of the microsphere manufacturing process. Lysozyme was chosen as a model biomacromolecule throughout this study. New information obtained from this study could help optimize the encapsulation of biomacromolecules of interest into the spongelike PLGA microspheres in a bespoke manner.

## 2. Materials and Methods

### 2.1. Materials

A poly-d,l-lactide-*co*-glycolide with a lactide:glycolide ratio of 75:25 (PLGA, inherent viscosity = 0.25 dL/g in chloroform; Tg = 41 °C) was obtained from Lakeshore Biomaterials, Inc. (Birmingham, AL, USA). Isopropyl formate with 98% purity was supplied from Alfa Aesar (Haverhill, MA, USA). A 28% ammonia solution was obtained by Merck Millipore (Darmstadt, Germany). Lysozyme human, fluorescein isothiocyanate-dextran (FITC-dextran, Mw = 40,000 g/mol), nile red (9-diethylamino-5H-benzo-α-phenoxazine-5-one), cetyltrimethylammonium bromide (CTAB), and Tween 80 were procured from Sigma-Aldrich (St. Louis, MO, USA). Micro bicinchoninic acid (BCA) reagents were purchased from Thermo Scientific (Waltham, MA, USA). Polyvinyl alcohol (PVA; 88% hydrolyzed, Mw = 25,000 g/mol) was from Polysciences, Inc. (Warrington, PA, USA). Dialysis membrane tubing (MWCO = 12–14 kD) was purchased from Spectrum Laboratories, Inc. (Rancho Dominguez, CA, USA). 

### 2.2. Preparation of Spongelike PLGA Microspheres

The ammonolysis-based single emulsion technique reported elsewhere was slightly modified to produce spongelike PLGA microspheres [27]. A dispersed phase consisting of 4 mL of isopropyl formate and 0.25 g of PLGA was emulsified in 40 mL of a 0.5% PVA solution using a digital hotplate stirrer. After 3 min stirring, the 28% ammonia solution (3 to 7 mL) was added to the oil-in-water emulsion. Ammonolysis of isopropyl formate, taking place in the dispersed phase, led to water-soluble isopropanol and formamide formation. As they served as anti-solvents toward PLGA, oil droplets were quickly transformed into solid microspheres. At the same time, their leaching into the aqueous phase resulted in numerous macropores across the microsphere matrices. After 30 min stirring, the microsphere suspension was poured into a dialysis membrane bag. After 8 h stirring in 1 L of distilled water, the microspheres were collected by filtration. These microspheres were either immediately used for lysozyme loading as described below (‘wet microspheres”) or vacuum-dried at ambient temperature (“dried microspheres”). Since both microspheres had an open pore structure, they were often referred to as open-pore microspheres in text.

### 2.3. Encapsulation of Lysozyme Into Spongelike Microspheres

**Figure 1:**
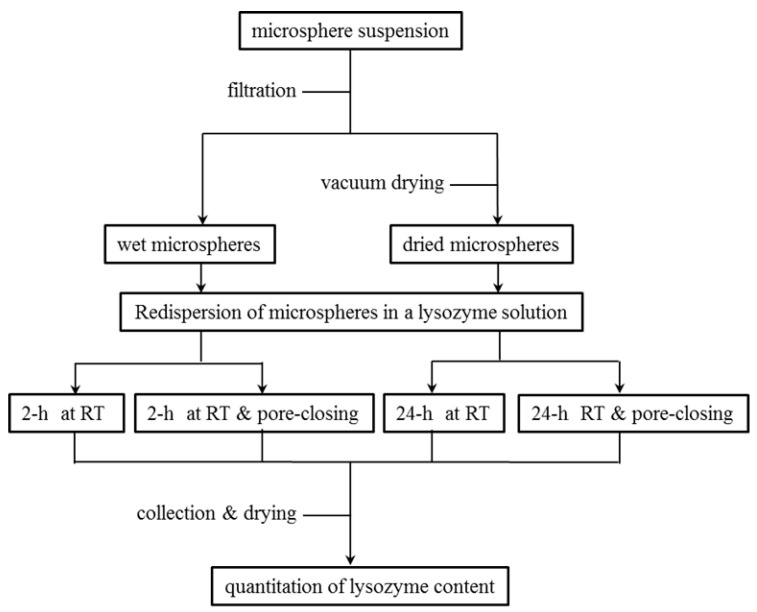
The schematic representation of various experimental settings for lysozyme loading into porous poly-d,l-lactide-co-glycolide (PLGA) microspheres. RT stands for room temperature.

Various experimental procedures were carried out to encapsulate lysozyme into porous PLGA microspheres. Typically, wet microspheres obtained at the final step of our microsphere manufacturing process were immediately mixed with an aqueous lysozyme solution containing 0.2% Tween 80 at RT (lysozyme concentration varied from 25 mg/mL to 200 mg/mL). The microsphere suspension was gently mixed using the SLRM-3 multimixer (Seoulin Bioscience Inc., Bundang-gu, Gyeonggi-do, Korea) and equilibrated for various periods (typically, 2 h). At the end of this process, the microspheres still maintained an open pore structure. In the practice of self-healing, the temperature of the aqueous phase was raised to 41 °C and left for 2 h (these microspheres that underwent this self-healing step were specified as either closed-pore microspheres or microspheres with a closed pore structure). Microspheres were then washed with distilled water, collected by filtration, and vacuum-dried overnight at ambient temperature. To investigate the effect of surfactant type on lysozyme encapsulation, Tween 80 was replaced with CTAB. The concentration of each surfactant varied from 0.2% to 2%. All the above experiments were repeated using dried microspheres, to determine whether dried microspheres would behave differently. Figure 1 shows the outline of various experimental settings for lysozyme encapsulation into wet microspheres and dried ones.

### 2.4. Determination of Lysozyme Content in PLGA Microspheres

Lysozyme contents in microspheres before and after self-healing were determined by the method reported elsewhere [28]. Briefly, microsphere samples (12.0–32.5 mg) were put into a test tube containing 2 mL of dimethyl sulfoxide, which was gently shaken for 30 min. Added into this solution was 10 mL of a 0.05 N NaOH solution containing 0.5% SDS. After being left to stand still for 1 h, an aliquot (100 mL) of the above solution was mixed with the BCA working solution (200 mL) in microplate wells. After 1 h incubation at 37 °C, the color intensity was measured at 562 nm by a microplate reader (model VERSAmax; Molecular Devices, San Jose, CA, USA). The lysozyme concentration of a sample was determined using a standard calibration curve made of a series of known lysozyme concentrations.

### 2.5. Scanning Electron Microscopy (SEM)

The morphology of PLGA microspheres was investigated by SEM (model JSM-5200; Jeol Korea Ltd., Seoul, Korea). The inside of spongelike PLGA microspheres was observed by two methods. They were sprinkled over a double-sided adhesive tape attached to a metal stub in a taping process. Another tape was overlaid onto it, pressed lightly, and removed. A part of the microsphere surface could be peeled off through this procedure, revealing its internal morphology. As an alternative method, microspheres were immobilized in 5 Minute^®^ Epoxy (Devcon^®^ home, Solon, OH, USA), hardened, and then sectioned with a metal blade. Since the taping method did not work toward closed-pore PLGA microspheres, they were mixed with the epoxy resin and cross-sectioned after hardening, as mentioned earlier. Before SEM observation, all microsphere samples were sputter-coated with platinum in an argon atmosphere.

### 2.6. Pore Size Distribution

The surface of porous PLGA microspheres was peeled off by using the taping method. After a circle with a radius of 10 mm at the center of the SEM micrograph was drawn, the sizes of pores inside the circle were measured using the ImageJ program (https://imagej.nih.gov/ij, the National Institutes of Health, Bethesda, MD, USA). Depending upon microsphere samples, a total of 1764–1959 pores were selected for measurement.

### 2.7. Encapsulation of Nile Red and FITC-Dextran into Porous PLGA Microspheres

Nile red was encapsulated into PLGA microspheres by the ammonolysis-based single emulsion technique. This process was carried out to stain the skeleton of our spongelike PLGA microspheres selectively. PLGA (0.25 g) and nile red (2.5 mg) were dissolved in 4 mL of isopropyl formate. This dispersed phase was emulsified in 40 mL of the 0.5% aqueous PVA solution. After stirring for 3 min, 3 mL of the ammonia solution was added into the o/w emulsion to harden emulsion droplets into porous PLGA microspheres. After 30 min stirring, the microsphere suspension was placed inside the dialysis membrane bag and was dialyzed in distilled water for 8 h. Microspheres were separated and equilibrated at RT for 2 h in 0.2% Tween 80 solution containing 10 mg/mL FITC-dextran. The microspheres were filtered, washed with distilled water, and dried under vacuum.

### 2.8. Confocal Laser Scanning Microscopy

The presence of nile red and FITC-dextran inside PLGA microspheres were visualized by a confocal laser scanning microscope (model LSM 880 with AiryScan; Carl Zeiss AG, Oberkochen, Germany). For image detection of nile red in the microspheres, 561 nm argon laser light was used to excite the dye, and fluorescence was collected through a 640 nm emission filter. Excitation and emission wavelengths used to detect FITC-dextran were 489 and 533 nm, respectively.

### 2.9. Statistical Analysis

An independent two-sample unpaired *t*-test was performed to compare the means of two groups. When a *p*-value was greater than 0.05, it was considered that there was no difference between the two groups’ means.

## 3. Results and Discussion

### 3.1. Spongelike Characteristics of Porous PLGA Microspheres

**Figure 2:**
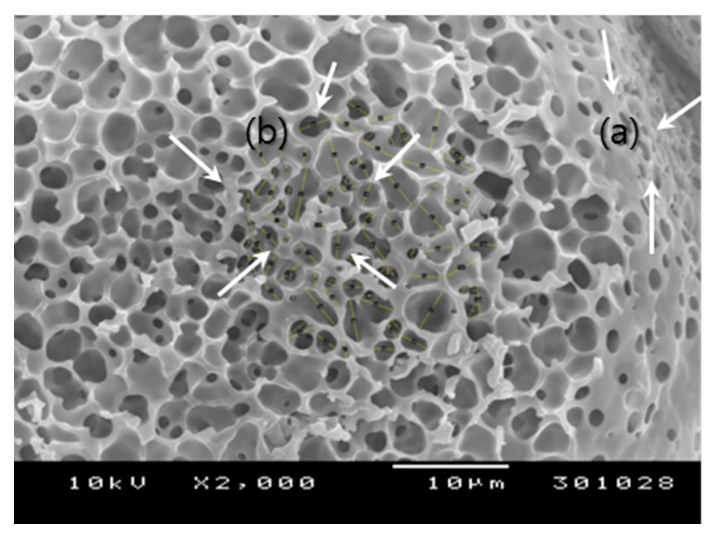
A SEM image showing (**a**) the surface and (**b**) internal morphology of a typical spongelike PLGA microsphere (bar size = 10 µm). As a part of the microsphere surface is peeled off, its inside is exposed without damaging its internal structure. The surface and inside of the microsphere have numerous pores that are inter-connected to have an open pore structure.

**Figure 3:**
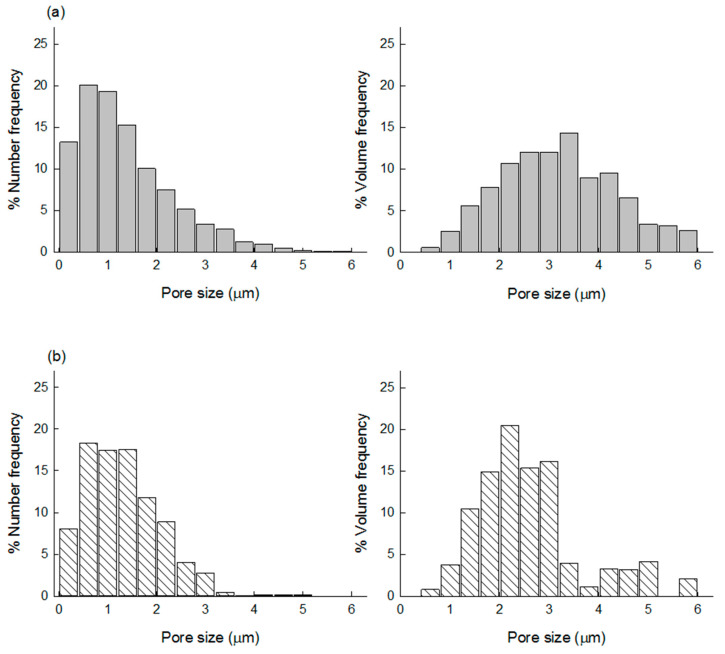
The pore size distributions of spongelike PLGA microspheres prepared using (**a**) 3 mL or (**b**) 7 mL of the ammonia solution. The microsphere porosity is determined by the amount of the ammonia solution used to trigger ammonolysis of isopropyl formate, the dispersed solvent of PLGA.

It is challenging to cut porous microspheres in a cross-section while retaining their internal structure. An appropriate technique should be developed to expose their intact inside. For instance, Zhang et al. used a cryogenic grinding method to reveal their porous PLGA microspheres [29]. In our case, the inside of our spongelike microspheres was readily displayed using the simple taping method. Since this method permitted the peeling off a part of the microsphere surface, it was possible to observe their surface and internal morphology at the same time (Figure 2). Figure 3 shows the pore size distributions of the spongelike microspheres determined by the NIH ImageJ program. So far, most porous PLGA microspheres have been frequently produced by w_1_/o/w_2_ double-emulsion methods using various types of porogens. By sharp contrast, our microencapsulation technique employed a porogen-free simple o/w emulsion technique using isopropyl formate, a non-halogenated ICH class 3 solvent. This new approach led to the formation of extremely porous PLGA microspheres resembling a spongy morphology (Figure 2). There were countless micron-sized pores/voids in the microsphere inside, and pores also were present ubiquitously on its surface. The mechanism for forming such spongelike porosity and the strategy of how to control microsphere porosity were discussed in detail elsewhere [27].

### 3.2. Lysozyme Loading into Open-Pore PLGA Microspheres

**Figure 4:**
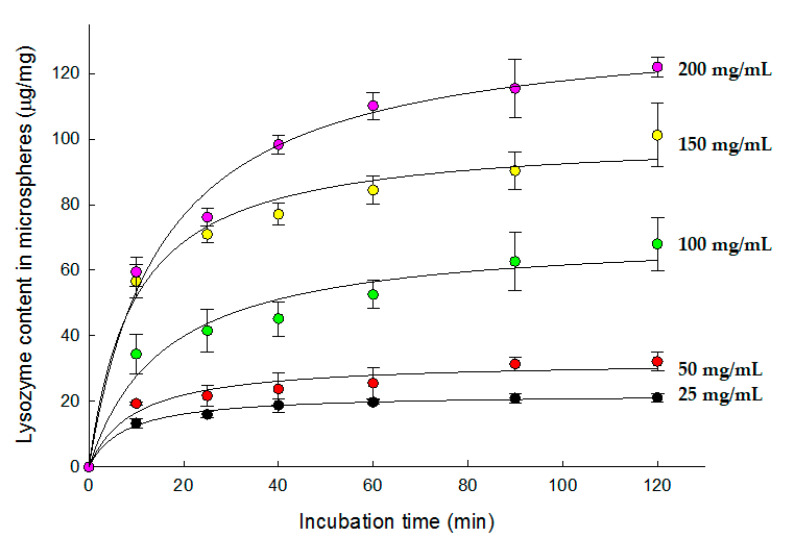
Lysozyme loading into wet PLGA microspheres prepared using 7 mL of the ammonia solution. They were incubated in aqueous 25 mg/mL to 200 mg/mL lysozyme solutions containing 0.2% Tween 80 for the specified periods of time. Lysozyme contents in the microspheres were then determined.

After wet microspheres were dispersed in aqueous 25–200 mg/mL lysozyme solutions at RT, the amounts of lysozyme in the microspheres as a function of time were determined. As a lysozyme concentration increased, more lysozyme was loaded into the porous microspheres (Figure 4). Under our experimental conditions, open-pore microspheres containing up to 12.2 ± 0.3% of lysozyme could be produced. The time for maximal lysozyme loading seemed to be 2 h at all concentrations. On the basis of these results, whenever wet microspheres were used for lysozyme loading, their incubation period in an aqueous lysozyme solution was fixed at 2 h. Considering that other previous studies set the equilibrium time at 24 to 72 h [20,21,23], it can be concluded that our wet microspheres reached equilibrium reasonably quickly.

**Figure 5:**
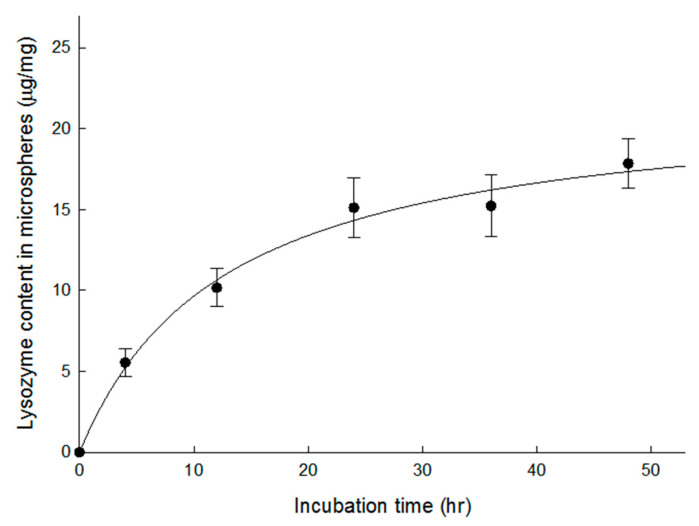
Lysozyme loading into dried microspheres prepared using 7 mL of the ammonia solution. They were dispersed in a 100 mg/mL lysozyme solution containing 0.2% Tween 80 at RT. At specified time intervals, lysozyme contents in the microspheres were measured.

Dried preformed PLGA microspheres were used in most previous studies for protein loading. Accordingly, dried microspheres were dispersed in an aqueous 100 mg/mL lysozyme solution containing 0.2% Tween 80 at RT. Lysozyme contents in the microspheres were measured as a function of time (Figure 5). After 24 h, the amount of lysozyme in the microspheres appeared to reach a plateau: its payload was 15.1 ± 1.9 µg/mg. Unexpectedly, this protein content was much lower than that observed with wet microspheres (Figure 4). When wet microspheres were incubated in the same aqueous lysozyme solution for only 2 h, its payload was 68.0 ± 8.1 µg/mg. Water occupying the pores of wet microspheres might have helped lysozyme molecules penetrate more easily from the external aqueous phase to the inside of the microspheres. On the contrary, it took a long time to sufficiently hydrate the pores inside dried microspheres, even with the aid of Tween 80. The difference in hydration degree between wet microspheres and dried ones seemed to influence lysozyme loading efficiency.

**Figure 6:**
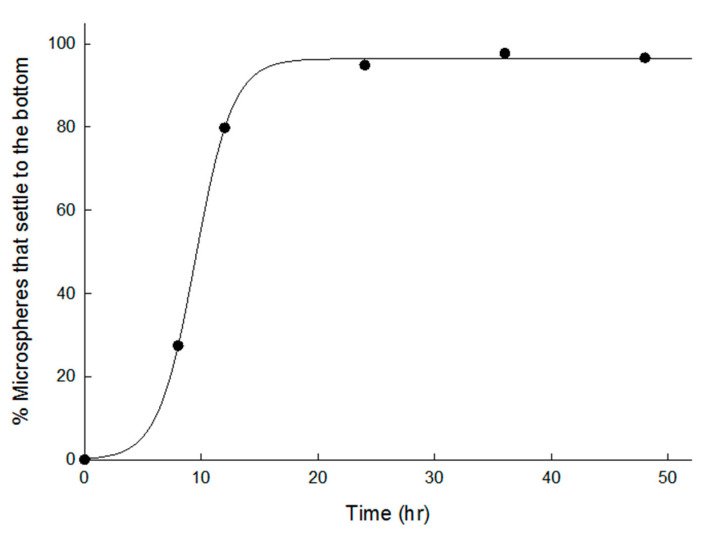
Sedimentation rates of dried microspheres prepared using 7 mL of the ammonia solution. They were dispersed in 0.2% Tween 80 solution at RT, and the amounts of their sediments were determined over time.

Once microspheres are sufficiently hydrated, they settle to the bottom of a container. To study the sedimentation rate of dried microspheres, they were put onto an Eppendorf tube containing an aqueous 0.2% Tween 80 solution. The percentage of microsphere sediments at the bottom of the Eppendorf tube was monitored as a function of time (Figure 6). Initially, most of the microspheres floated on the solution due to their spongelike skeleton. After 24 h, all the microspheres settled to the bottom of the Eppendorf tube. These data and those in Figure 5 suggest that an incubation time to sufficiently hydrate dried microspheres is crucial when they are put to use for protein loading. It is often presumed that a biomacromolecule solution can penetrate easily into porous PLGA microspheres and fill macrovoids inside their porous matrix. However, since they do not pass well in reality, measures to maximize their permeability (e.g., incubating for sufficient time, applying vacuum or pressure, or inducing active penetration) are required. Our study demonstrates that using wet microspheres with apparent porosity is an excellent alternative to encapsulate protein efficiently.

**Figure 7:**
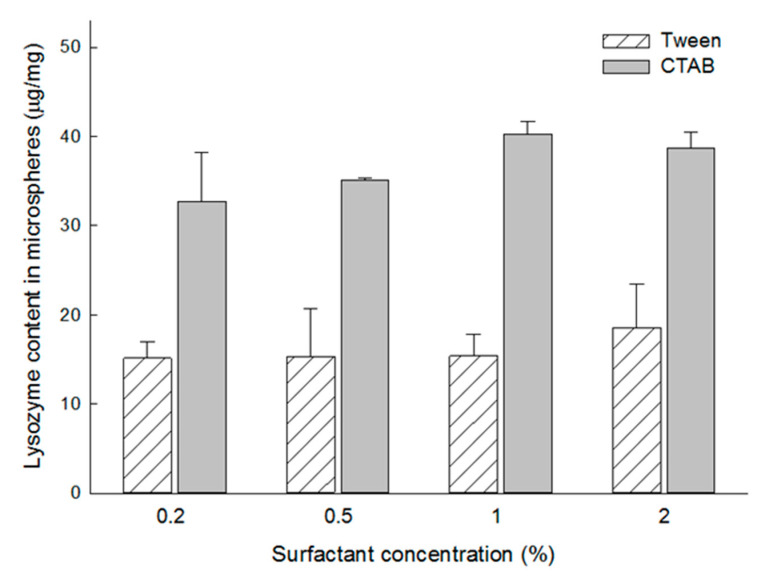
Effect of surfactant type upon lysozyme loading in dried microspheres prepared using 7 mL of the ammonia solution. In terms of lysozyme loading efficiency, CTAB shows a superior functionality than Tween 80.

Tween 80 is frequently used to facilitate water ingress into porous hydrophobic matrices of PLGA microspheres. Our study has raised a question as to whether the type of surfactant could affect the wetting degree of our porous PLGA microspheres and, consequently, lysozyme payload. Therefore, dried microspheres were incubated at RT for 24 h in a 100 mg/mL lysozyme solution containing 0.2% to 2% Tween 80 or CTAB. It was statistically demonstrated that lysozyme loading was favored with CTAB over Tween 80 at all concentrations (the highest *p*-value of 0.008 was observed at the 0.2% concentration). CTAB increased the extent of lysozyme loading more than twice compared to Tween 80 (Figure 7).

### 3.3. Comparison of Lysozyme Loadings before and after Self-Healing

**Figure 8:**
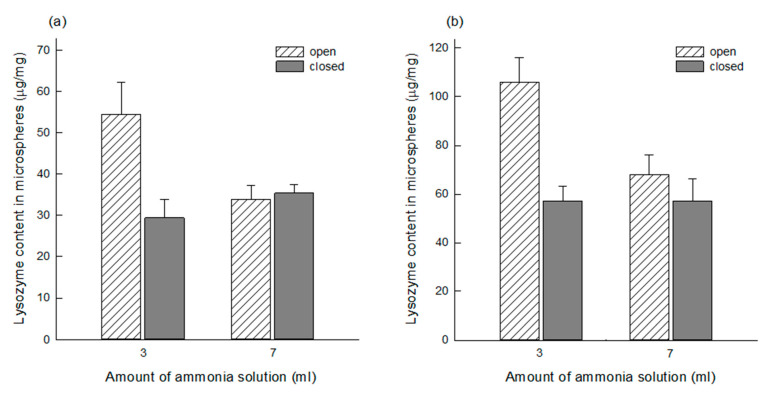
Comparison of lysozyme payloads in porous microspheres before and after pore closure. Wet microspheres prepared using 3 mL and 7 mL of the ammonia solution were equilibrated at RT for 2 h in (**a**) 50 mg/mL and (**b**) 100 mg/mL lysozyme solutions containing 0.2% Tween 80, which was further subjected to self-healing. Lysozyme contents in the microspheres were determined before and after self-healing.

Before self-healing, PLGA microspheres had a porous network in which countless pores were interconnected (Figure 2). While wet PLGA microspheres maintained the open pore structure, a more considerable amount of lysozyme was loaded into the microspheres produced using 3 mL of the ammonia solution than those made by 7 mL of the ammonia solution. The corresponding *p*-values of the data shown in Figure 8a,b were 1.15 × 10^−10^ and 2.43 × 10^−11^. This result might have arisen from their difference in pore characteristics, as shown in Figure 3. After self-healing, the microsphere morphology changed from an open pore structure to a closed pore structure. Not only the closed-pore microsphere had a non-porous compact surface, but also they lost a porous network. It was questioned whether there was any change in lysozyme content in wet microspheres after self-healing. It was expected that, since pore-closing might force water inside microsphere pores to squeeze out, a portion of lysozyme in the microspheres would leach into an aqueous phase. Toward wet PLGA microspheres prepared using 3 mL of the ammonia solution, self-healing caused a reduction in about 40% of lysozyme in the microspheres (Figure 8). However, the above supposition was disproportionate when wet microspheres prepared using 7 mL of the ammonia solution were initially used for lysozyme loading. Interestingly, lysozyme of similar amounts remained in both closed-pore microspheres prepared using 3 and 7 mL of the ammonia solution. For example, a *p*-value of 0.97 was obtained when their means shown in Figure 8b were compared by the *t*-test.

### 3.4. Effect of Surfactant Type upon Lysozyme Loading into Dried Microspheres

**Figure 9:**
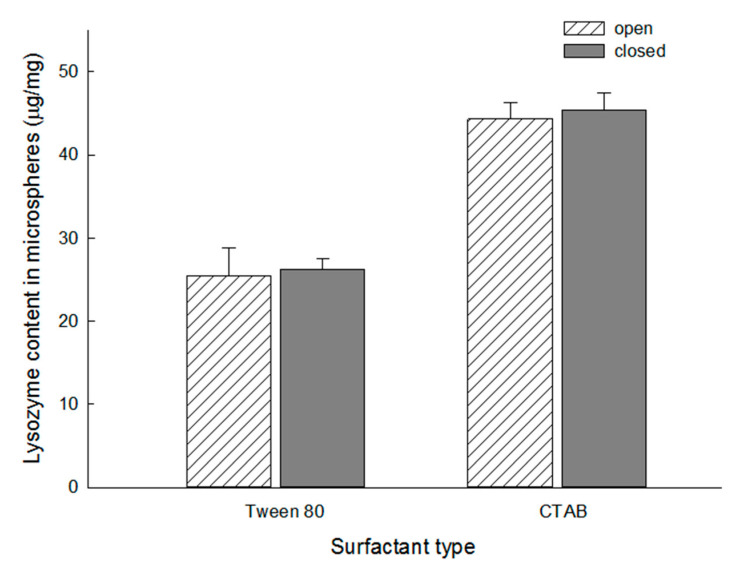
Effect of surfactant type upon lysozyme payloads in PLGA microspheres before and after pore closure. Dried microspheres prepared using 3 mL of the ammonia solution were first incubated in a 100 mg/mL lysozyme solution containing 0.2% either Tween 80 or CTAB (RT/2 h), and self-sealing (41 °C/2 h) was carried out. Lysozyme contents in microspheres before and after self-healing were determined.

As pointed out earlier, dried microspheres with an open pore structure showed a lower efficiency of lysozyme loading than open-pore wet microspheres (Figure 4 and Figure 5). After dried microspheres were dispersed in a 100 mg/mL lysozyme solution containing 0.2% either Tween 80 or CTAB at RT for 24 h and followed by self-healing, the amounts of lysozyme in the closed-pore microspheres were determined (Figure 9). There was no statistically significant difference in lysozyme contents before and after self-healing. Regardless of which surfactant was used, the same conclusion was reached. The *p*-values of CTAB-treated microspheres and Tween 80-treated ones were 0.34 and 0.60, respectively. Our findings contrast to a previous report that the encapsulation efficiency of bovine serum albumin into PELA microcapsules after self-healing was 45.2% [22]. Besides, the type of surfactant affected the loading efficiency of lysozyme to a great extent. Substituting Tween 80 with CTAB caused significant improvement in lysozyme payload in closed-pore microspheres from 26.2 ± 1.4 to 45.3 ± 2.1 µg/mg.

**Figure 10:**
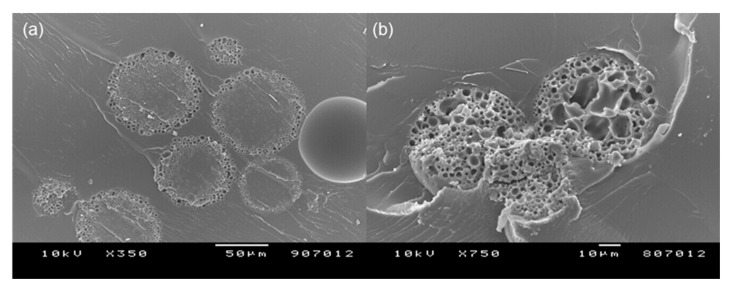
SEM images of the internal morphology of closed-pore PLGA microspheres prepared using 7 mL of the ammonia solution. Dried microspheres were first equilibrated in an aqueous solution containing 0.2% either (**a**) Tween 80 or (**b**) CTAB, and were then subjected to self-healing.

It is necessary to elaborate on why the type of surfactant affects the level of lysozyme loading. Modulating protein adsorption by surfactants has been an essential issue in the pharmaceutical industry. Tween 80 is often used to prevent protein adsorption on many hydrophobic surfaces. Because the surfactant has a much greater surface activity than protein, it preemptively occupies the hydrophobic surface [30]. Hoffmann et al. investigated the interaction between lysozyme and Tween 80 by isothermal titration calorimetry [31]. They concluded that the interaction and binding of Tween 80 to lysozyme were negligible. By contrast, the positively charged head and carbon alkyl chain of the CTAB were suggested to interact strongly with lysozyme through electrostatic and hydrophobic interactions [32]. Fang also proposed a method of complexing CTAB and an antigen to maximize the antigen adsorption on the PLGA microsphere surface [33]. Based on these results, it can be suggested that the lysozyme loading efficiency is improved as the CTAB-lysozyme complex adsorbs much better onto porous PLGA microspheres. However, in this study, our effort was focused on investigating how the type of surfactant (CTAB vs. Tween 80) influenced the pore structure of PLGA microspheres during self-healing. For this experiment, dried microspheres were first incubated in each surfactant solution at RT for 2 h and then followed by the pore-closing step (41 °C for 2 h). Figure 10 shows the SEM images of the closed-pore microspheres in a cross-section. Surprisingly, when the equilibrating medium contained Tween 80, even though some pores were present in the proximity of the microsphere surface, countless pores inside the original microspheres disappeared. Overall, their inside was transformed to become a compact matrix. In contrast, self-healing with CTAB brought up the formation of new larger voids, and the resultant microspheres had a closed pore structure in which pores were no longer interconnected.

**Figure 11:**
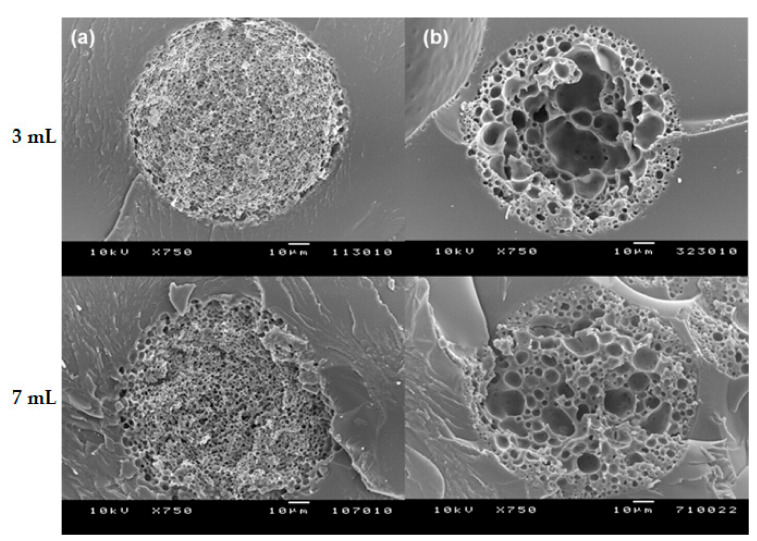
SEM images of the internal morphology of various PLGA microspheres (**a**) before and (**b**) after self-healing (bar size = 10 µm). Wet microspheres prepared using 3 mL and 7 mL of the ammonia solution were first incubated in 0.2% Tween 80 solution at RT for 2 h and were followed by self-healing.

**Figure 12:**
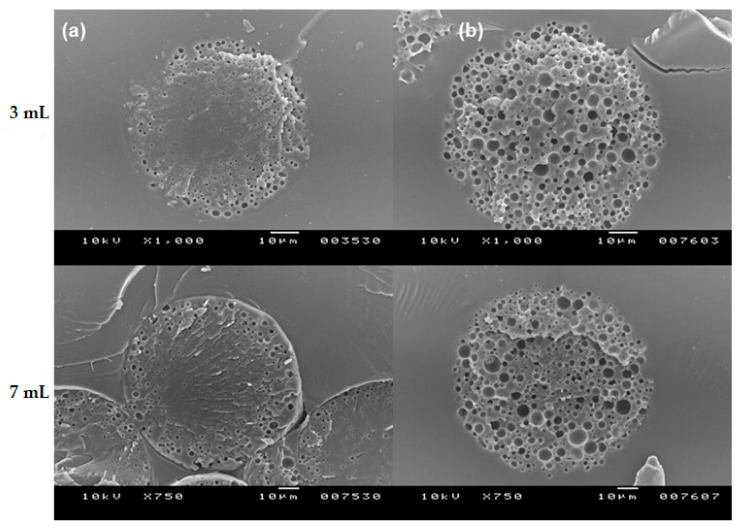
SEM images of the internal morphology of dried PLGA microspheres after self-healing (bar size = 10 µm). Dried microspheres prepared using 3 mL and 7 mL of the ammonia solution were first incubated at RT for (**a**) 2 h and (**b**) 24 h and were followed by self-healing.

Based on this result, it is speculated that the hydration degree of porous PLGA microspheres has a significant influence on the rearrangements of polymer chains during the self-healing process. As CTAB showed a superior wetting functionality than Tween 80, more water existed in the spongelike pores of the microspheres. Water molecules were likely to interfere with the polymer behavior during self-healing, thereby affecting the microstructure of closed-pore microspheres (Figure 10). As a result, the microspheres self-healed with CTAB had a considerably higher volume of inner empty voids than those self-healed with Tween 80. If this argument is plausible, self-healing of wet microspheres in the Tween 80 solution should produce the microspheres of which their internal morphology is similar to that shown in Figure 10b (this prediction can be made because they are already sufficiently hydrated). When wet microspheres incubated in 0.2% Tween 80 solution for only 2 h were followed by self-healing, their inside morphology was in agreement with the expected morphology (Figure 11). The validity of our scientific reason was proved once again below. The results shown in Figure 6 demonstrated that dried microspheres dispersed in 0.2% Tween 80 solution at RT for 2 h were hardly hydrated, but their wetting degree significantly increased after 24 h. Therefore, if self-healing is performed toward the dried microspheres incubated for 24 h rather than 2 h, the number of macropores inside the microspheres would increase. The results of Figure 12 are consistent with this prediction. Previously, Reinhold and Schwendeman emphasized the dependence of self-healing upon microsphere porosity, PLGA end-capping, and the temperature of an aqueous protein solution [21]. Our study demonstrates that the hydration degree of microspheres plays a significant role in their pore structure determined by self-healing, which plays a decisive role in the efficiency of lysozyme loading.

### 3.5. Confocal Laser Scanning Microscopy

**Figure 13:**
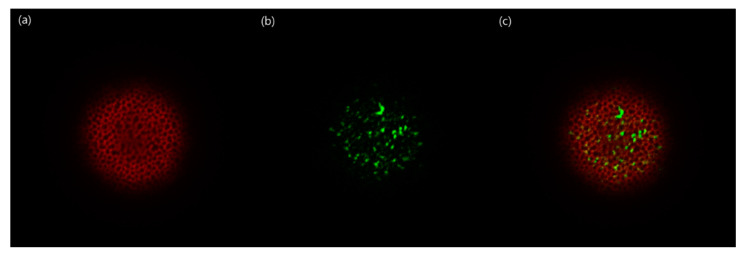
Confocal microscopic images showing the distributions of (**a**) nile red, (**b**) FITC-dextran, and (**c**) nile red and FITC-dextran within PLGA microspheres.

Confocal images of PLGA microspheres are shown in Figure 13. Nile red, a hydrophobic dye exhibiting red fluorescence, was well encapsulated in the microsphere matrix. Referring to the SEM image shown in Figure 2, it is understood that the blackened areas in the microspheres were voids of the spongelike microsphere. Because FITC-dextran is hydrophilic, it stayed in the voids of the microsphere and fluoresced green. PLGA microspheres containing both nile red and FITC-dextran exhibited red and green fluorescence images. These results support that a hydrophilic biomacromolecule was encapsulated therein after preparing spongelike microspheres containing a hydrophobic drug in advance. Closing the pores on the surface of the microspheres could provide a long-acting depot system for multiple drugs with contrasting physical attributes.

## 4. Conclusions

The PLGA microspheres produced by the ammonolysis-based microencapsulation process are structurally different from typically reported porous PLGA microspheres. They have a sponge matrix in which numerous pores are evenly distributed and interconnected to form a porous network. This feature might be advantageous to protein loading through self-healing. Several parameters of the self-healing microencapsulation process governed the lysozyme loading efficiency. Relevant examples included lysozyme concentration in an aqueous medium, incubation time, surfactant type, pore characteristics of microspheres (e.g., porosity, open-pore/closed-pore structure), and microsphere hydration degree. So far, dried preformed microspheres have been commonly used to load biomacromolecules, but wet microspheres obtained at the end of the microsphere manufacturing process were preferred in this study. Using the wet microspheres without drying shortened the incubation time to hydrate microspheres and substantially improved lysozyme loading efficiency. Besides, the state of microsphere hydration greatly influenced the microstructure of the closed-pore microspheres after self-healing. Understanding all these critical process parameters would help encapsulate a variety of biomacromolecules into the spongelike PLGA microspheres. Biomacromolecule–microsphere interactions and their release characteristics would also be worth exploring in future studies.

## Data Availability

Our data are contained within the article. They also are available on request from the corresponding author.
